# Supplementary material for: Probing the spinor nature of electronic states in nanosize non-collinear magnets
Source: Nat Commun. 2016 Oct 10;7:13000. doi: 10.1038/ncomms13000 (PMC5062547; doi:10.1038/ncomms13000)
Supplement: Supplementary Information — Supplementary Figures 1-10, Supplementary Notes 1-11 and Supplementary References [file ncomms13000-s1.pdf]

Supplementary Figure 1

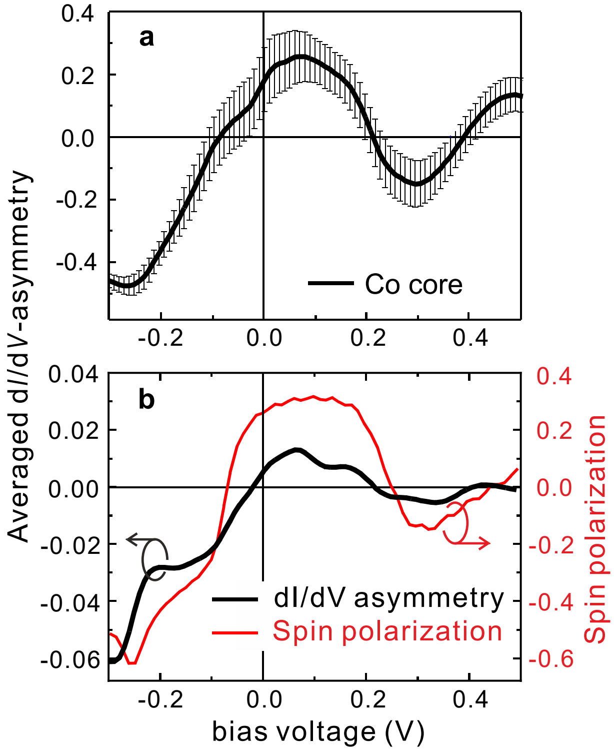

Tip properties probed in reference Co nanostructures. a,**b**, Differential conductance asymmetry curves (black) as a function of bias voltage $A_{dI/dV}$. The experimental curve in **a** is obtained in the Co core of the Fe|Co island (for the region enclosed by black dotted curve in Fig. 2f). The experimental curve in **b** is obtained for the pure Co island in Ref. [1]. The red curve in **b** presents the result of first-principle calculation of the spin polarization of a Co bilayer island at the tip position (Ref. [1]).

Supplementary Figure 2

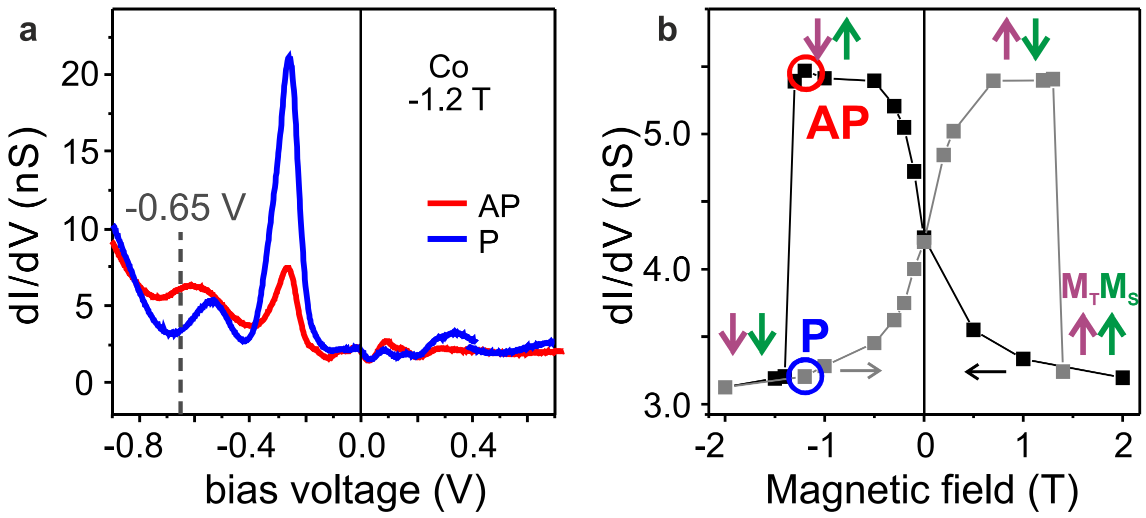

In-field spin-polarized scanning tunneling spectroscopy. **a** Differential conductance measured at the center of the Co core of the Fe|Co island shown in Fig. 2**f** with antiparallel (AP, red) and parallel (P, blue) magnetization configurations between tip and sample. **b** Magnetic hysteresis loops of differential conductance dI/dV signals (V_b =$-$0.65 V) of the STS spectra measured at the center of the Co core of the island shown in Fig. 2**f** (field sweep between $-$2.0 T and +2.0 T). Gray (black) color code denotes the dI/dV values measured for the forward (backward) field sweep. The magenta and green arrows indicate the magnetization directions of the tip and Co core, respectively, for different magnetic states.

Supplementary Figure 3

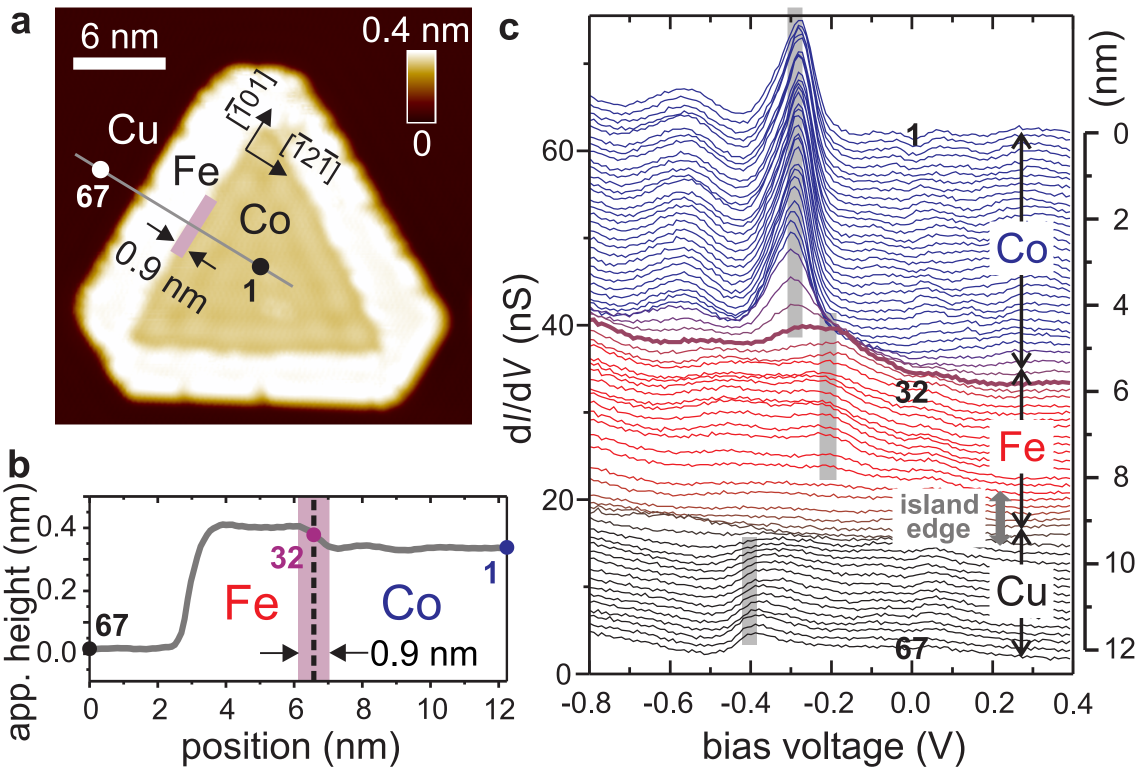

Spatially resolved STS across the Fe-Co border in a Fe|Co island. **a** STM image of the Fe|Co nanoisland presented in Fig. 2**f** ($V_b = 0.5$ V, $I_t = 1$ nA). **b** STM apparent height profile from position 1 to position 67 along the line in **a**. The index 32 and the dotted line indicate the topographic center of the Fe-Co border. The purple bar depicts the electronic transition width of 0.9 ± 0.1 nm (gray bar) determined from the dI/dV spectra analysis in **c**. **c** 67 differential conductance spectra from the Co center (top) through Fe stripe to the Cu substrate (bottom) region, taken at a spacing of 0.18 nm, measured along the gray line depicted in **a** (STS stabilization: V_{stab} = 0.5 V and I_{stab} = 1 nA). The ruler on the right side identifies the distance of the measurement position of each spectrum from that of spectrum 1. The gray bars indicate the characteristic peak energies of the spectra measured in the Co (−0.31 V), Fe (−0.22 V), and Cu (−0.4 V) regions.

Supplementary Figure 4

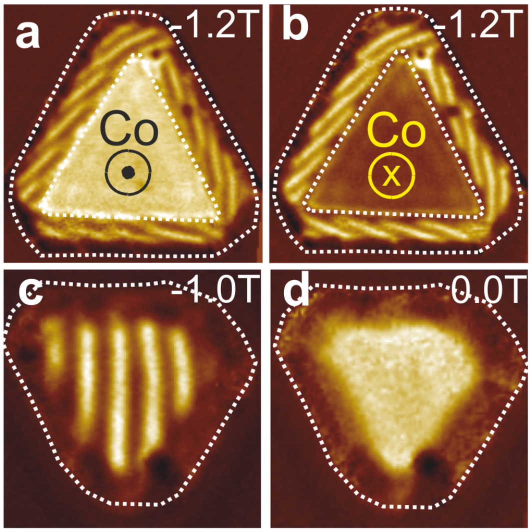

Differential conductance maps of Fe|Co and pure Fe nanoislands. a,b Differential conductance maps of the Fe|Co island in Fig.2 **f** for the (**a**) antiparallel (AP) and (**b**) parallel (P) state (V_b = −0.65 V and −1.2 T). The out-of-plane magnetization directions for the Co core are represented by ⊙ (out-of-the-plane) in **a** and ⊗ (into-the-plane) in **b**. **c,d** Differential conductance maps of the pure Fe island in Fig. 2**a** for (**c**) Measurement at −1.0 T and (**d**) at 0 T (V_b = −0.65 V). The island boundaries are indicated by the white dotted curves. The boundary is inferred from the spatially resolved dI/dV spectroscopy shown in the Supplementary Note 3 and in Supplementary Fig. 3.

Supplementary Figure 5

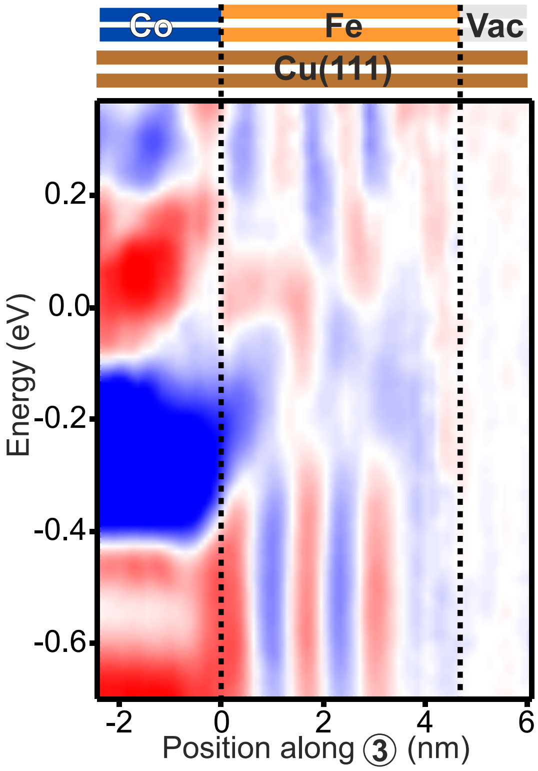

Energy resolved dI/dV asymmetry of Fe|Co island. Energy resolved dI/dV asymmetry line scan for the Fe|Co island along direction 3, indicated in Fig. 2**g**.

Supplementary Figure 6

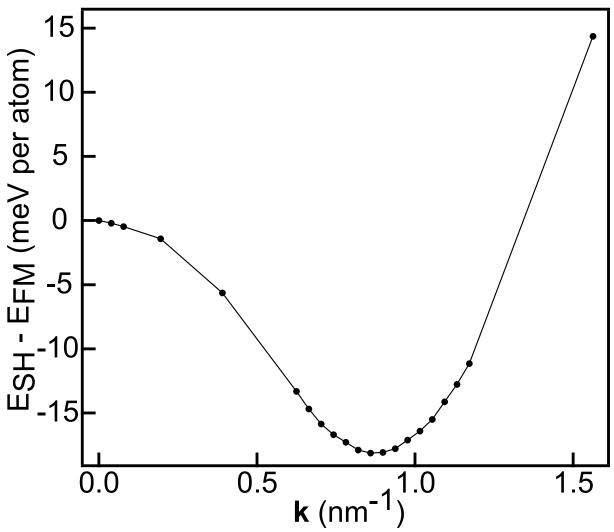

Calculated spin helix formation energy for an extended bilayer Fe film. Calculated energy difference between a helical magnetic order and a ferromagnetic order as a function of k_{SH} along the direction of the k_{SH} observed in the experiment. The atomic structure of the calculation model is a free-standing bilayer Fe with inter-atomic distances mimicking Cu(111) epitaxial arrangement with topmost Fe in bridge-site stacking.

Supplementary Figure 7

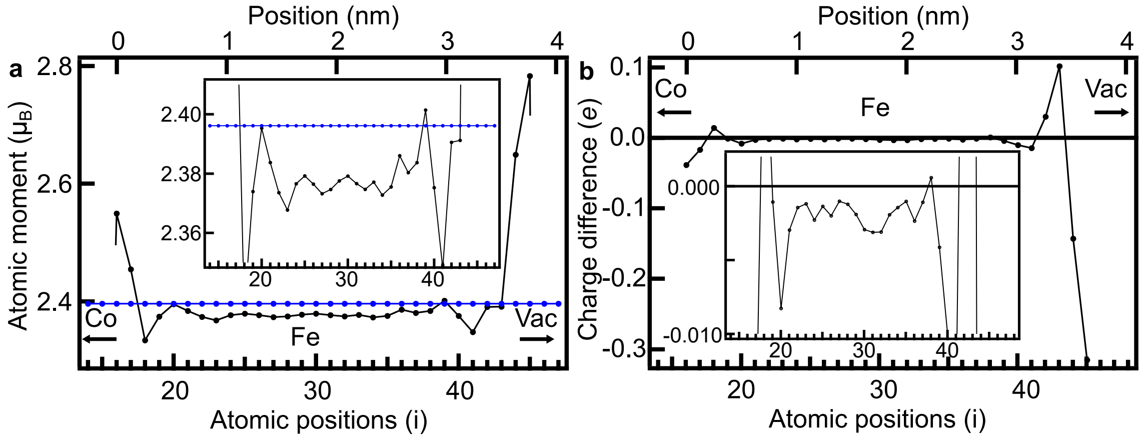

Calculated atomic moments and electronic charge differences in the Co/Fe/Vac system. a DFT-based calculation of the atomic moments of the Fe atoms for the 16 Co / 30 Fe / 8 Vac system (black data points). Reference data for an infinite Fe bilayer with ideal spin helix are given by blue data points.**b** Calculated difference in electronic charges in the Fe atoms of the 16 Co / 30 Fe / 8 Vac system as compared to the infinite bilayer Fe with ideal spin helix. The numbering of the Fe atoms from i = 17 to 46 reflect the numbering given in Fig. 3a of the main text.

Supplementary Figure 8

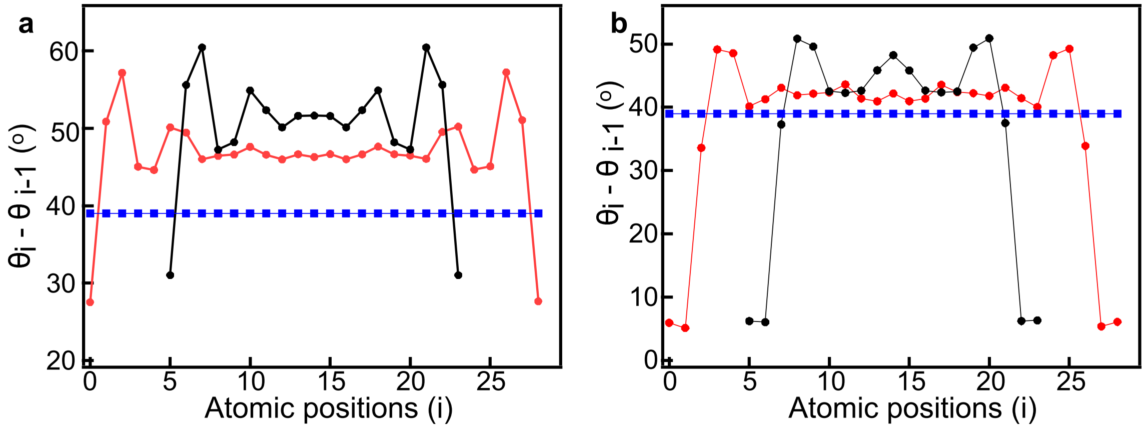

Calculation in bilayer Fe stripes systems with symmetric boundaries. a,**b** DFT-based calculation of the angle between the atomic magnetization directions of neighboring atoms along bilayer Fe stripes with the 20 Fe (black) and 30 Fe (red) atoms, confined between 8 Co (**a**) atoms or 8 vacuum spheres (**b**), respectively. The data for 20 Fe atom stripe are shifted horizontally by 5 atomic positions for better visualization. The blue data points give the angle between neighboring Fe atoms in an ideal spin helix, as shown in Fig. 3**c**.

Supplementary Figure 9

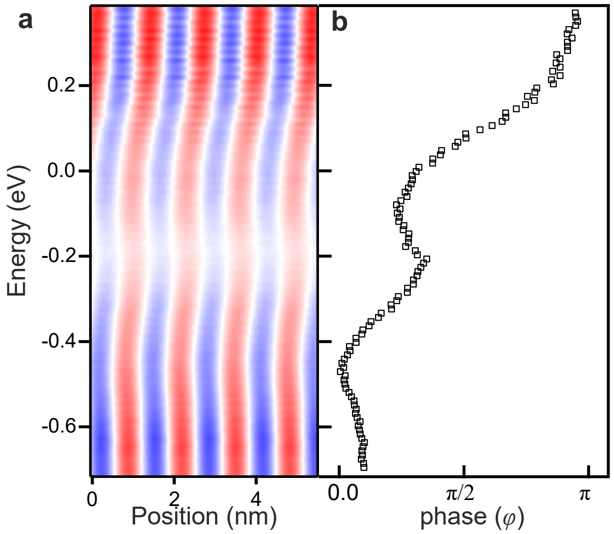

Fit of the energy dependent dI/dV asymmetry data of the pure Fe island. a resulting dI/dV asymmetry as given by the fit. **b** energy dependence phase angle (φ) used for the fit in **a**.

Supplementary Figure 10

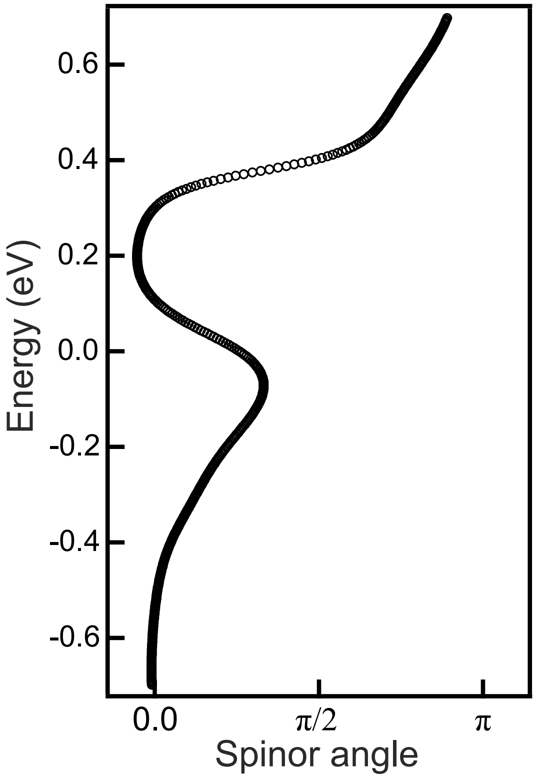

Calculated energy dependence of the spinor angle of symmetrically confined Fe bilayer. Calculated energy dependence of the spinor angle of the 8 Vac / 30 Fe / 8 Vac system. The spinor angle is defined as the angle between the electronic magnetization direction calculated 4 Å above the surface (as probed in sp-STM/S experiments) and the atomic moment direction in the Fe atom in the sample surface. The continuous variation of the spinor angle with energy is the hallmark of the non-collinearity between the electronic magnetization and the atomic moment.

Supplementary Note 1

Tip spin polarization. In Supplementary Fig. 1, we compare three curves carrying similar physical information. Two of them are the experimental differential conductance asymmetries as a function of the bias. Both experimental curves are obtained on the bilayer Co samples. The curve in Supplementary Fig. 1a corresponds to the Co core of the Fe|Co island studied in the present paper. The Co core area is marked in Fig. 2f by black dotted circle. The curve in Supplementary Fig. 1b is obtained on the pure Co island in Ref. [1]. Further experimental comparison can be found in Ref. [2]. The third curve (red curve in Supplementary Fig. 1b) presents the result of the first-principles calculation of the spin-polarization of the Co bilayer at the tip position [1]. The similar shapes of all three curves allow us to conclude that the spin polarization of the tip is almost constant and maintains its magnetization orientation out-of-plane in the energy interval studied in the paper.

Supplementary Note 2

Differential conductance d*I*/d*V* hysteresis curve of a ferromagnetic Co island core measured by sp-STM. We perform sp-STM/S measurements with a Co/Cr-coated W tip in an external magnetic field oriented normal to the sample surface to study the differential conductance signal as a function of bias voltage between tip and sample. A positive bias indicates electron flow from the tip towards the sample. Supplementary Figure 2a shows differential conductance (dI/dV) spectra measured at the center of the Co core of the Fe$|$Co island, see Fig. 2f of the main text, at -1.2 T with antiparallel (AP - red) and parallel (P - blue) magnetization configurations between tip and sample. Supplementary Figure 2b shows the magnetic hysteresis loop of the differential conductance signal measured at V_b = -0.65 V at the same position for a field sweep between -2.0 T and $+2.0$ T. The sharp signal change of the hysteresis curve reveals the switching field [3] of the Co core at $\pm$ 1.25 T. The curve shows a superparamagnetic tip [4] response with a saturation field of $\pm$ 1.0 T. The hysteresis curve identifies the field value of -1.2 T to ensure AP and P alignments of sample and tip magnetization directions. Measurements of the dI/dV maps of pure Fe islands (as described in the main text) were obtained with a well characterized tip, which has a similar response. The dI/dV maps were taken at -1.0 T, see Fig. 2c, and at 0 T (Supplementary Fig. 4d).

Supplementary Note 3

Spatially resolved d*I*/d*V* spectra between Co and Fe in a Fe|Co island. Supplementary Figure 3a shows a constant current STM image of the Fe|Co island identified in Fig. 2f. We discriminate three elements (Co, Fe, and Cu) by differences in the apparent heights, as indicated in the line scan shown in Supplementary Fig. 3b. The chemical identification is done spectroscopically by local STS measurements. Supplementary Fig. 3c shows a spatial sequence of dI/dV spectra from the center of the Co core to the Cu substrate, along the line shown in grey in Supplementary Fig. 3a. The peak at $V_b \approx -0.3$ V identifies the Co 3d$_{z2}$-minority state of bilayer Co on Cu(111) [5]. The broad peak at $V_b \approx -0.2$ V identifies the bilayer Fe structure in bridge-site-stacking of the topmost Fe atoms [6–8]. The step-like feature at -0.4 V identifies the Cu surface state [9]. We observe a gradual change over 0.9 nm from the Co spectra to Fe spectra across the Co-Fe interface. We define the transition region width as the spatial interval between the last pure Co spectrum and the first pure Fe spectrum. We find a transition region width of 0.9 ± 0.1 nm. This corresponds to less than 4 atomic distances in Cu(111), and this is compatible with an atomically sharp interface.

Supplementary Note 4

Differential conductance maps of Fe|Co and pure Fe nanoislands. Supplementary Figures 4a and b (Supplementary Fig. 4c and d) show the dI/dV maps of the Fe|Co island (pure Fe island) with AP and P magnetization (in-field and zero-field) configurations, respectively, at $\mu_0 H = -1.2$ T (-1.0 T) at a bias voltage $V_b = -0.65$ V. These maps were used to calculate the dI/dV asymmetry maps shown in Fig. 2b,g using Equations 2 and 3, as explained in the main text.

Supplementary Note 5

Energy resolved d*I*/d*V* asymmetry of the Fe|Co island. In Fig. 2h of the main text we present the energy resolved dI/dV asymmetry along direction 2 of the Fe|Co island of Fig. 2f. Supplementary Figure 5 shows it for direction ③, see Fig. 2g . Both asymmetry maps are very similar revealing that the discussed features are of general nature.

Supplementary Note 6

Calculations for ideal helices. Here we briefly discuss the results of the calculations for an infinite Fe bilayer. Supplementary Figure 6 shows the energy of the spin helix (SH) as a function of the wave vector of the helix. The energy scale is chosen with reference to the ferromagnetic (FM) state (wave vector $k = 0$). The energy minimum is at $\mathbf{k} \approx 0.86$ nm^{-1}. This identifies an incommensurate spin helix as the magnetic ground state. The corresponding angle between the spin moments of neighboring atoms has a constant value of about 39°. Although our method of the calculations (see Methods section) is different from the method employed in Ref. 8 the calculated $\mathbf{k}$-dependences of the energy are very similar, revealing the robustness of the physical picture.

It is important to realize that the very existence of the helical spin structures is intimately related to the generalized translational symmetry of the system [10]: The system is invariant with respect to the combination of usual space translations R$_n$ and pure spin rotations by angles $\mathbf{q}{\cdot}\mathbf{R_n}$. If the effects of magnetic anisotropy caused by the spin-orbit coupling (SOC) are negligible, as it is the case here, the operators of generalized translations commute with the Hamiltonian of the spin helix. This leads to the equivalence of atoms of the ideal helix. This generalized periodicity allows to reduce, in a mathematically exact manner, the calculation of an incommensurate helix to the consideration of a small chemical unit cell instead of large magnetic super cell.

The atomic potentials and magnetic structure of the ideal helix were used as starting potentials and magnetic structure for the calculations of the laterally confined samples. Already the first iteration leads to distortion of the helical structure and difference of atomic potentials of different Fe atoms.

Supplementary Note 7

Atomic moment and deficiency of atomic charge in the distorted spin helix. In Supplementary Fig. 7, we present the results of the calculations for the Fe atomic moments and the Fe electronic charges for the spin helix of the infinite Fe bilayer and for the 16 Co / 30 Fe / 8 Vac system. Here, the numbers give the number of atomic spheres in a bilayer arrangement, which are positioned to mimic the epitaxial order on Cu(111) with topmost Fe in bridge-site-stacking [8]. In the proximity to interfaces we obtain strong irregular deviations of the values of atomic moments and electronic charges difference of 16 Co / 30 Fe / 8 Vac system as compared to the corresponding values for the spin helix. In the central part of the Fe region the deviations are smaller (see insets of Supplementary Fig. 7). However, as expected on the basis of symmetry arguments, they present and reflect the influence of the lateral confinement on the part of the Fe stripe that is distant from the interfaces.

Supplementary Note 8

Distortions in symmetrically confined systems. In Supplementary Fig. 8, we present the results of the calculations for the systems with symmetric interfaces and different sizes of the Fe bilayer: Vac/20Fe/Vac (black) and Vac/30Fe/Vac (red) (a), Co/20Fe/Co (black) and Co/30Fe/Co (red) (b), where the numbers give the number of atomic spheres in a bilayer arrangement, which are positioned to mimic the epitaxial order on Cu(111) with topmost Fe in bridge-site-stacking [8]. In all cases we observe a significant deviation of the magnetization orientation from that of an ideal spin helix, where the largest deviations occur near the interfaces. The values for an ideal helix are given by the blue data points for comparison. The angular variation near the central parts of the larger Fe sample with 30 Fe atoms deviate less from the value of the ideal helix as compared to that of the smaller Fe sample with 20 Fe atoms. This aspect is regarded as a nanosize effect, which still awaits experimental scrutinization in future studies. There is also a pronounced difference between samples with vacuum (a) and Co (b) interfaces. These calculations reveal an interesting interplay between the nanosize and proximity effects. The resulting magnetic structure depends on both the width of the Fe stripe and the interface. Interfaces with vacuum or Co influence the magnetic structure differently. These effects are also clearly observed in the calculations presented in the main text of the paper (see Fig. 3a).

Supplementary Note 9

Fitting of the energy dependent dI/dV asymmetry maps. In the discussion in the main text of the physical effects reflecting the spinor nature of electronic states, we fitted the experimental data of the energy resolved dI/dV asymmetry map (Fig. 4) in order to extract quantitative information regarding the spinor angle. Here, we explain the fitting analysis.

The dI/dV signal measured with sp-STM is described, in a generalized theory of Tersoff and Hamann [11, 12], as

$$dI/dV \propto n_T n_S + \mathbf{m}_{\mathrm{tip}}.\mathbf{m}_{\mathrm{el}}. \tag{1}$$

Where n_T and n_S are the local density of states (LDOS) of the tip and of the sample at the tip apex position, and $\mathbf{m}_{\mathrm{tip}}$ and $\mathbf{m}_{\mathrm{el}}$ are the local magnetization of the tip and the sample at the tip apex position [1], respectively.

Since the dot-product ($\mathbf{m}_{\mathrm{tip}}.\mathbf{m}_{\mathrm{el}}$) is proportional to the projection of the $\mathbf{m}_{\mathrm{el}}$ on the fixed direction of the tip magnetization, the differential conductance asymmetry calculated on the basis of Supplementary Equation 1 is determined by the value of this projection. The experiment provides the energy and spatial dependence of the asymmetry and, therefore, with the energy and spatial dependence of the projection of $\mathbf{m}_{\mathrm{el}}$ on the direction of the tip magnetization selected as the global z-axis.

If we assume that the direction of $\mathbf{m}_{\mathrm{el}}$ at a given spatial position x is collinear to the underlying atomic moment, in the case of ideal spin helix we must expect that the spatial variation of $\mathbf{m}_{\mathrm{el,z(x)}}$ will be defined by a cosine function of the type $A\cos(kx+\varphi)$, where the x direction is the direction of the helix propagation and **k** is the wave vector of the helix. Indeed, in the case of large Fe sample, the spatial dependence of the experimental differential asymmetry can be well described by the cosine function with energy-independent **k**. However, for an ideal spin helix, the angle φ must also be energy independent. However, this property is not satisfied

for the laterally confined samples. Therefore, for each energy E the spatial dependence of the differential asymmetry was approximated with the function $A(E)\cos\left(kx + \varphi(E)\right)$. The angle $\varphi(E)$ obtained in the fitting are shown in Fig. 4 and Supplementary Fig. 9.

Supplementary Note 10

Spinor states in non-collinear magnetic structures. In a NCM, the electronic Kohn-Sham Hamiltonian does not commute with the operator of spin projection on any selected axis. This necessitates to represent the electronic wave functions in the form of a two-component spinor $\psi(\mathbf{r}) = \begin{pmatrix} \psi_\uparrow(\mathbf{r}) \\ \psi_\downarrow(\mathbf{r}) \end{pmatrix}$ and the electronic potential in the form of a two-by-two matrix. We emphasize that this spinor form of the electronic wave functions is not related to the SOC, which is neglected in our consideration, but it is solely due to the non-collinearity of the magnetic structure. Assuming a spherically symmetric form of the atomic potentials the effective potential in the i-th atomic sphere is written in the form

$$\mathbf{V}_i(r) = \mathbf{U}(\mathbf{e}_i)^\dagger \begin{pmatrix} V_i^+(r) & 0 \\ 0 & V_i^-(r) \end{pmatrix} \mathbf{U}(\mathbf{e}_i) \tag{2}$$

where the vector $\mathbf{e}_i$ determines the direction of the local spin quantization axis of the i-th atom coinciding with the direction of the atomic moment. In the local spin-coordinate system the potential has a spin diagonal form; V_i^+ and V_i^- are the spin-up and spin-down potentials in the local atomic system; $\mathbf{U}((\mathbf{e}_i)$ is the matrix of spin-$\frac{1}{2}$ rotation transforming the potential from the atomic system of the ith atom to the global system.

The contribution of an electronic state to the magnetization is given by the vector function $\mathbf{m}(\mathbf{r}) = \begin{pmatrix} \psi_\uparrow(\mathbf{r}) & \psi_\downarrow(\mathbf{r}) \end{pmatrix}^* \sigma \begin{pmatrix} \psi_\uparrow(\mathbf{r}) \\ \psi_\downarrow(\mathbf{r}) \end{pmatrix}$ where σ is the vector of the Pauli matrices. We emphasize that for a spinor function with nonzero spin-up and spin-down components there is a nonzero magnetization component orthogonal to the quantization axis since the expectation values of the x and y components of the magnetization are given by expressions $\mathbf{m}_x(\mathbf{r}) = Re[\psi_\uparrow(\mathbf{r})^* \psi_\downarrow(\mathbf{r})], \mathbf{m}_y(\mathbf{r}) = Im[\psi_\uparrow(\mathbf{r})^* \psi_\downarrow(\mathbf{r})]$. This is an important difference of the electronic states in NCM compared to the states of collinear magnets.

To calculate the spinor angle for a given atomic or empty (vacuum) sphere we integrate the vector spin density at a given energy over the volume of the sphere. The spinor angle is the

angle that this integral vector forms with the local quantization axis parallel to the corresponding atomic moment. As discussed in the text of the paper and Supplementary Note 6 for an ideal helix the calculated angle is always either 0 or 180°, and it does not contain information about spin-mixing of the electronic states. On the other hand, for distorted magnetic structures the angle is energy dependent and can assume arbitrary values providing information that is closely related to the spinor characteristics of the electronic states.

Next we consider an infinite perfect spin helix with arbitrary wave vector. Here the states cannot be characterized by a given spin projection and must be considered as two-component spinors with both components nonzero. The generalized translations keep the atoms equivalent and it is sufficient to consider one atom. Without loss of generality we can assume that the atomic spin moment of the atom 0 is parallel to the z axis. The magnetization of each of these states will not be collinear to the atomic magnetization. However, the ideal exchange helix is invariant under the transformation combining the spatial reflection in the zy-plane and pure spin rotation by 180 degrees about z axis. The direction of the spin helix propagation is parallel to the x-axis. If we act with this operation on an arbitrary state of the system $\psi(r)$, the new spinor function will also be an eigenstate of the Hamiltonian of the ideal spiral, corresponding to the same energy. The m_x and m_z spin components of the spin moment in the atomic sphere 0 will be opposite. Therefore at any energy, the contributions to the m_x and m_z components of the atomic moment are zero, and the spin magnetization at any energy is exactly collinear to the atomic moment.

In the case of the low-symmetry distorted magnetic configuration (Fig. 1c), the atomic moments also are non-collinear and the states, like in ideal spin helix, are also spinors with non-zero transverse components of the magnetization. However, in this case there is no symmetry-determined compensation of the transverse components of different states. Therefore, at each energy the interplay of the contributions of individual states is different. This results in different

deviation of the electronic magnetization from the atomic quantization axis.

Supplementary Note 11

Spinor angle in pure Fe island. It is now instructive to come back to Fig. 2c and to remark that also in this case, which we treated as an approximation of an ideal spin helix, we observe signatures of the influence of the nanoscale size of the sample. The transition between positive (red) to negative (blue) $\mathrm{d}I/\mathrm{d}V$ asymmetry gives rise to a slightly curved stripe contrast as a function of energy in Fig. 2c, whereas the calculation give a strictly straight stripe contrast in Fig. 3a.

In order to extract quantitative information of the curved stripe contrast in the Fig. 2c, we perform the same analysis (fit by a function $A_i \cos(kx + \varphi_i)$) as discussed in the main text and Supplementary Note 8. Supplementary Fig. 9a shows the resulting fit for the energy dependent $\mathrm{d}I/\mathrm{d}V$ asymmetry in Fig. 2c, describing the bilayer Fe case. A visual comparison of Supplementary Fig. 9a and Fig. 2c shows a good agreement between the fit and the experimental data. Supplementary Fig. 9b reveal the energy dependence of the phase angle φ, respectively. The phase plot (Supplementary Fig. 9b) reveals a continuous variation of the angle φ, which is ascribed to the spinor angle. We also calculated the energy dependence of the angle between electronic magnetization and atomic moment for the a representative Fe atom in the Vac/Fe/Vac sample. Supplementary Fig. 10 shows the spinor angle (defined in the main text) as a function of the energy. We observe a continuous variation, which resembles the phase (Supplementary Fig. 9b). The combination of this experimental and theoretical results indicate that also in the case of an almost ideal spin helix, the electronic magnetization direction probed at the tip position is not exactly collinear to the atomic moment of the sample at the position underneath the tip. Although the distortions of the spin helix are relatively small for this sample, they are present. The physical origin of the effect is exactly the same as in the case of the Co/Fe/Vac sample: it is the spinor nature of the electronic states in the system with broken symmetry.

Supplementary references

[1] Oka, H. *et al.* Spin-dependent quantum interference within a single magnetic nanostructure. *Science* **327**, 843–846 (2010). URL http://www.sciencemag.org/content/327/5967/843.abstract.

[2] Corbetta, M. *et al.* Magnetic response and spin polarization of bulk Cr tips for in-field spin-polarized scanning tunneling microscopy. *Japanese Journal of Applied Physics* **51**, 030208 (2012). URL http://stacks.iop.org/1347-4065/51/i=3R/a=030208.

[3] Ouazi, S. *et al.* Magnetization reversal of individual Co nanoislands. *Phys. Rev. Lett.* **108**, 107206 (2012). URL http://link.aps.org/doi/10.1103/PhysRevLett.108.107206.

[4] Phark, S.-h., Fischer, J. A., Corbetta, M., Sander, D. & Kirschner, J. Superparamagnetic response of Fe-coated W tips in spin-polarized scanning tunneling microscopy. *Applied Physics Letters* **103**, 032407 (2013). URL http://scitation.aip.org/content/aip/journal/apl/103/3/10.1063/1.4815993.

[5] Diekhöner, L. *et al.* Surface states of cobalt nanoislands on Cu(111). *Phys. Rev. Lett.* **90**, 236801 (2003). URL http://link.aps.org/doi/10.1103/PhysRevLett.90.236801.

[6] Biedermann, A., Rupp, W., Schmid, M. & Varga, P. Coexistence of fcc- and bcc-like crystal structures in ultrathin Fe films grown on Cu(111). *Phys. Rev. B* **73**, 165418 (2006). URL http://link.aps.org/doi/10.1103/PhysRevB.73.165418.

[7] Gerhard, L. *et al.* Magnetoelectric coupling at metal surfaces. *Nat Nano* **5**, 792–797 (2010). URL http://dx.doi.org/10.1038/nnano.2010.214.

[8] Phark, S. H. *et al.* Reduced-dimensionality-induced helimagnetism in iron nanoislands. *Nat Commun* **5**, 5183 (2014). URL http://dx.doi.org/10.1038/ncomms6183.

[9] Crommie, M. F., Lutz, C. P. & Eigler, D. M. Imaging standing waves in a two-dimensional electron gas. *Nature* **363**, 524–527 (1993). URL http://dx.doi.org/10.1038/363524a0.

[10] Sandratskii, L. M. Noncollinear magnetism in itinerant-electron systems: Theory and applications. *Advances in Physics* **47**, 91–160 (1998). URL http://www.tandfonline.com/doi/abs/10.1080/000187398243573.

[11] Tersoff, J. & Hamann, D. R. Theory and application for the scanning tunneling microscope. *Phys. Rev. Lett.* **50**, 1998–2001 (1983). URL http://link.aps.org/doi/10.1103/PhysRevLett.50.1998.

[12] Wortmann, D., Heinze, S., Kurz, P., Bihlmayer, G. & Blügel, S. Resolving complex atomic-scale spin structures by spin-polarized scanning tunneling microscopy. *Phys. Rev. Lett.* **86**, 4132–4135 (2001). URL http://link.aps.org/doi/10.1103/PhysRevLett.86.4132.
